# Improving time-to-result: head-to-head comparison of three rapid AST systems for Gram-negative bacteremia, including the newly developed VITEK REVEAL

**DOI:** 10.1128/jcm.01050-25

**Published:** 2025-10-09

**Authors:** Damiano Squitieri, Giulia Menchinelli, Carlotta Magrì, Tiziana D’Inzeo, Barbara Fiori, Margherita Cacaci, Maurizio Sanguinetti, Giulia De Angelis, Brunella Posteraro

**Affiliations:** 1 Dipartimento di Scienze Biotecnologiche di Base, Cliniche Intensivologiche e Perioperatorie, Università Cattolica del Sacro Cuore 96983 , Rome, Italy; 2 Dipartimento di Scienze di Laboratorio ed Ematologiche, Fondazione Policlinico Universitario A. Gemelli IRCCS, Rome, Italy; 3 Unità Operativa “Medicina di Precisione in Microbiologia Clinica”, Direzione Scientifica, Fondazione Policlinico Universitario A. Gemelli IRCCS, Rome, Italy; Mayo Clinic, Baltimore, Maryland, USA

**Keywords:** Gram-negative bacteria, blood cultures, rapid antimicrobial susceptibility testing, minimum inhibitory concentration, time to result, volatile organic compounds

## Abstract

Early antimicrobial susceptibility testing (AST) in Gram-negative (GN) bloodstream infections is critical to guide appropriate therapy. This study evaluated three rapid AST (RAST) systems—VITEK REVEAL, direct-from-blood-culture VITEK 2 (VITEK 2-RAST), and EUCAST disk diffusion (DD-RAST)—using 220 prospectively collected GN-positive blood cultures (GN-PBCs). A total of 18 GN species were tested, including *Enterobacterales*, *Acinetobacter baumannii*, and *Pseudomonas aeruginosa*, against 25 antibiotics, four of which were next-generation β-lactam/β-lactamase inhibitor (BL/BLI) antibiotics. Reference broth microdilution was used for comparison. The VITEK REVEAL assay showed 97.1% essential agreement (EA), −7.7 bias, and 98.3% categorical agreement (CA) across 3,603 organism/antibiotic combinations. The VITEK 2-RAST assay showed 96.2% EA, −10.4 bias, and 98.4% CA across 3,941 combinations. The DD-RAST assay showed 98.2% CA across 2,388 combinations. Among 164 GN-PBCs tested with all three assays, EA rates were 97.5% for VITEK REVEAL and 96.3% for VITEK 2-RAST, with bias values of −7.1 and −6.9, respectively. Across all three assays, the CA rate was ≥98.2%, and very major error (VME) rates were ≤1.8%. Mean time-to-result (TTR) was significantly shorter with VITEK REVEAL (6 h 32 min) compared to VITEK 2-RAST (13 h 51 min) and the fixed 8 h of DD-RAST. In resistant organisms, VITEK REVEAL produced significantly faster results than in susceptible or intermediate ones, including BL/BLI antibiotics. *A. baumannii* yielded the shortest TTR, with no observed VME. These results support the use of RAST directly on GN-PBCs, with VITEK REVEAL emerging as a particularly promising option for rapid and accurate testing.

**IMPORTANCE:**

This study is the first to conduct a direct comparison of three rapid antimicrobial susceptibility testing (RAST) systems—VITEK REVEAL, VITEK 2-RAST, and DD-RAST—on a large, prospectively collected cohort of Gram-negative-positive blood cultures (GN-PBCs). The data offer valuable insights for laboratories evaluating RAST implementation, especially in EUCAST-based contexts. Among the systems tested, VITEK REVEAL stood out for its combination of rapid turnaround and high accuracy, including for antibiotic-resistant organisms and β-lactam/β-lactamase inhibitor (BL/BLI) antibiotics. These findings underscore the potential of RAST systems to deliver timely and reliable susceptibility results directly from GN-PBCs, thereby supporting more effective antimicrobial stewardship and clinical decision-making. However, the real-world impact will also depend on how well these systems integrate into routine workflows, as seamless implementation can directly affect time-to-result (TTR)—an essential element in optimizing the management of GN-bloodstream infection (BSI).

## INTRODUCTION

Bloodstream infections (BSIs) caused by Gram-negative (GN) bacteria (GN-BSIs) carry a high risk of morbidity and mortality, particularly when effective antimicrobial therapy is not administered promptly (1, 2). In patients with septic shock, each hour of delay in receiving appropriate antimicrobials is associated with a measurable increase in mortality (3). Even in less severely ill patients, delayed initiation of effective therapy has been linked to prolonged hospital stays and worse clinical outcomes (4, 5). Phenotypic rapid antimicrobial susceptibility testing (RAST) performed directly from positive blood cultures (PBCs) is therefore essential to guide timely and appropriate therapy, reduce the use of unnecessary broad-spectrum antimicrobials, and support antimicrobial stewardship efforts (6).

Several commercial systems, recently introduced to allow RAST reporting (typically within 8 h or less from the time of blood culture positivity), rely on technologies that accelerate phenotypic antimicrobial susceptibility testing (AST) beyond the pace of conventional subculture-based AST assays and then enable faster results directly from PBCs (7). Systems reviewed by MacVane and Dwivedi in 2024 (6) include ASTar (Q-linea, Uppsala, Sweden), LifeScale (Affinity Biosensors, Santa Barbara, CA, USA), Next-Generation Phenotyping (Selux, Charlestown, MA, USA), PhenoTest BC (Accelerate Diagnostics, Tucson, AZ, USA), and VITEK REVEAL (bioMérieux, Marcy l’Étoile, France), which all gained US-Food and Drug Administration (FDA) clearance. The VITEK REVEAL system represents a novel approach based on the detection of volatile organic compounds (VOCs) released during bacterial metabolism (8). It uses 96-well broth microdilution (BMD) plates to generate minimum inhibitory concentration (MIC) values in approximately 5.5–6 h. Recent evaluations in GN bacteremia have demonstrated excellent agreement with conventional AST assays (Sensititre [Thermo Fisher Scientific, Waltham, MA, USA], VITEK 2 [bioMérieux], or MicroScan WalkAway [Siemens Healthcare Diagnostics, Erlangen, Germany]) (9–11). The use of VOC sensing enables the system to deliver rapid results while maintaining conceptual similarity with reference BMD assays, which remain the gold standard for MIC determination (7).

RAST from GN-PBCs may also be performed using the VITEK 2 system directly (i.e., bypassing subculture) or the EUCAST direct inoculation of disk diffusion plates, which provides zone diameters interpretable at 4, 6, and 8 h (12, 13). These approaches are already embedded in many clinical BSI workflows, as in our laboratory (14, 15), and may be considered practical, lower-cost solutions that warrant further large-scale evaluations.

The increasing prevalence of antimicrobial resistance among GN pathogens further complicates empirical therapy selection and clinical management (16). Notably, the time required to obtain AST results may vary according to the resistance profile of the pathogen. In a recent study by Ostermann et al. (11) using VITEK REVEAL, a significant inverse correlation between time-to-result (TTR) and resistance prevalence was observed (−0.018 h for every 1% increase in resistance rate; *P* < 0.0001), suggesting that resistant organisms may yield faster results in some RAST systems. In contrast, MacVane et al. (17) reported a 1-day longer TTR for next-generation antimicrobial agents in patients with difficult-to-treat resistant phenotypes and carbapenem-non-susceptible organisms. This delay was attributed to conventional workflows involving reflexive testing—where next-generation agents are tested only upon detection of resistance to first-line agents, rather than being included in primary AST panels/cards. While efficient in terms of resources, such cascade testing may inadvertently delay final results for novel antimicrobial agents, underscoring the value of RAST systems/assays capable of providing broad-spectrum, rapid susceptibility data without relying on stepwise testing strategies.

In this study, we performed a comparative evaluation of three RAST assays—VITEK REVEAL, direct-from-blood-culture VITEK 2 (VITEK 2-RAST), and EUCAST rapid disk diffusion (DD-RAST)—on GN-PBCs. Results from each assay were assessed against the EUCAST reference BMD performed on subculture-derived isolates. Emphasis was placed on TTR, particularly for next-generation β-lactam/β-lactamase inhibitor (BL/BLI) antibiotics and antimicrobial-resistant organisms. In selected cases showing discordant results between VITEK REVEAL and the reference BMD assay, population analysis profile (PAP) experiments were performed to investigate potential heteroresistance.

## MATERIALS AND METHODS

### Study setting, clinical samples, and antimicrobial agents

**Fig 1:**
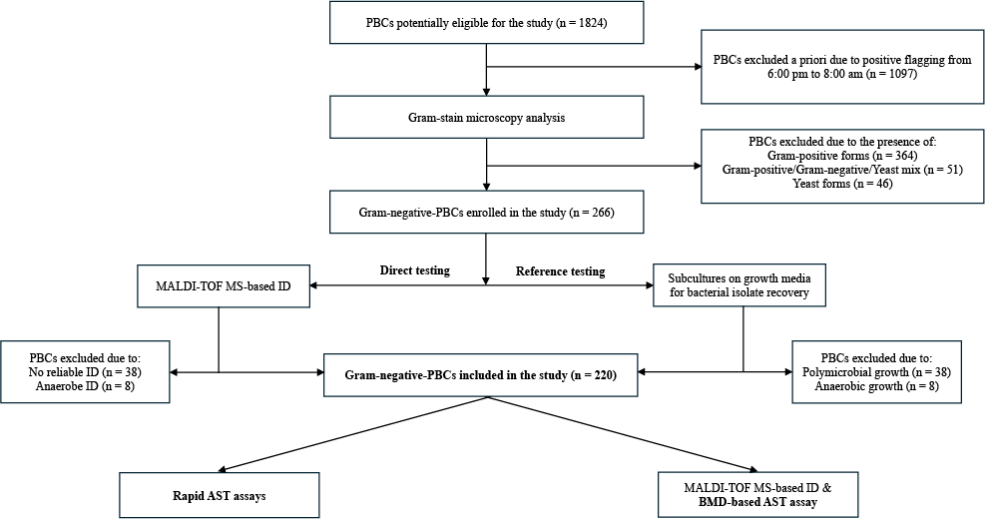
Of the 220 GN-PBC samples included in the study, all underwent direct antimicrobial susceptibility testing by at least one of the following assays—VITEK 2-RAST, VITEK REVEAL, or DD-RAST—depending on species-specific eligibility for each system. In parallel, bacterial isolates recovered from the same GN-PBC samples underwent matrix-assisted laser desorption ionization-time of flight mass spectrometry (MALDI-TOF MS)-based identification and reference BMD testing. AST, antimicrobial susceptibility testing; BMD, broth microdilution; ID, identification; PBC, positive blood culture.

This study was conducted at the clinical microbiology laboratory of Fondazione Policlinico Universitario A. Gemelli IRCCS, a large tertiary-care teaching hospital in Rome, Italy. The study protocol is outlined in Fig. 1. Of the 17,525 clinical blood cultures processed during the study period (January 2024 to June 2024), 3,083 (17.6%) were positive for bacterial growth, and 922 of these (29.9%) yielded GN organisms. Only GN-PBCs from unique patients were included.

PBC samples were obtained from blood culture (BC) bottles (BacT/Alert FA Plus [aerobic] and FN Plus [anaerobic], bioMérieux) after incubation in the BacT/Alert Virtuo automated BC system (bioMérieux) and subsequent positivity. For each positive BC, the first aerobic or anaerobic bottle that flagged positive and was available for microbiological testing was selected. Samples were processed Monday to Friday between 8:00 a.m. and 6:00 p.m., ensuring timely execution and reading of all RAST assays under standardized conditions. This schedule also allowed testing to occur before bacterial overgrowth (i.e., <10^9^ CFU/mL), which is particularly important for DD-RAST given its short incubation time and use of a non-standardized inoculum (13). Furthermore, it ensured that VITEK REVEAL testing was performed well within the 16-h window recommended by the manufacturer (9–11).

All eligible PBC samples underwent Gram staining, and only monomicrobial GN-PBCs were included. Species-level identification was performed using a direct matrix-assisted laser desorption ionization-time of flight (MALDI-TOF) mass spectrometry-based assay (Bruker Daltonics, Bremen, Germany), as previously described (18). Aliquots from each bottle were used both for direct RAST assays and for parallel subculture on MacConkey and 5% sheep blood tryptic soy agar (bioMérieux). If MALDI-TOF identification failed or mixed growth was observed on agar, the sample was excluded. Colonies obtained from overnight subculture were then used both for the reference BMD AST assay and to confirm species identification by MALDI-TOF mass spectrometry.

Details on the antimicrobial agents and their concentrations tested in each assay, as well as the bacterial species/antimicrobial agent combinations tested in the three RAST assays and in the EUCAST reference BMD assay (in which all combinations were tested), are provided in the supplemental material (Tables S1 and S2). In total, 18 GN bacterial species were tested, including 13 *Enterobacterales* and five non-*Enterobacterales*. Seven species were the most represented (≥8 organisms): *Escherichia coli* (*n* = 74), *Klebsiella pneumoniae* (*n* = 54), *Pseudomonas aeruginosa* (*n* = 22), *Proteus mirabilis* (*n* = 16), *Acinetobacter baumannii* (*n* = 14), *Klebsiella oxytoca* (*n* = 9), and *Serratia marcescens* (*n* = 8).

### VITEK REVEAL assay

The RAST assay using the VITEK REVEAL system was performed with 25 µL of each GN-PBC sample, which was directly diluted 1:1,000 in Pluronic water (Beckman Coulter, Brea, CA, USA), without prior removal of blood cells. Then, 115 µL of the diluted sample was inoculated into the wells of a VITEK REVEAL GN01-AST plate, which includes a panel of antibiotics targeting GN bacteria (Table S1). Each AST plate was covered with a proprietary sensor panel composed of an array of seven colorimetric chemical sensors positioned over each well and sealed using the dedicated plate sealer (Beckman Coulter). Plates were barcode-labeled and loaded into the VITEK REVEAL instrument, where they were incubated at 37°C with continuous agitation and imaged every 10 min. Colorimetric changes in the sensor array, induced by VOCs released during bacterial metabolism, allowed the system to determine MIC values in real time. One well on each plate served as a growth control (growth medium, no antibiotic), and another served as a negative control (no growth medium). Bacterial identification, obtained as previously described, was entered into the system interface to enable MIC interpretation. The assay protocol followed the procedures detailed in our validation study (19) and was consistent with those reported by other groups (9–11), in accordance with the manufacturer’s instructions.

### VITEK 2-RAST assay

The RAST assay using the VITEK 2 system was performed using AST-N438, AST-XN26, and AST-N440 cards, with the first two suitable for *Enterobacterales* and the third suitable for non-fermentative species (e.g., *P. aeruginosa*). These cards include a predefined series of antimicrobial agents at various concentrations and enable MIC determination through turbidimetric monitoring of bacterial growth (20). The protocol was adapted from previously published procedures (14, 21, 22), with minor modifications to allow direct testing of GN-PBC samples. Briefly, a fixed volume (5 mL) of each GN-PBC sample was transferred into a BD Vacutainer serum separator tube (BD, Franklin Lakes, NJ, USA) and centrifuged at 3,500 × *g* for 15 min. The resulting bacterial pellet was resuspended in sterile saline (bioMérieux) and adjusted to a turbidity of 0.5 McFarland, measured in a VITEK 2 cuvette. This suspension was then used to inoculate the appropriate AST card, which was incubated in the VITEK 2 instrument. MIC values and interpretive categories were automatically assigned using the Advanced Expert System (v9.02; bioMérieux).

### DD-RAST assay

The DD-RAST assay was performed according to the method developed by EUCAST for direct AST from PBCs (23). This method has been specifically validated for four GN bacterial species—*E*. *coli*, *K. pneumoniae*, *P. aeruginosa*, and *A. baumannii*—and is intended for use with aerobic BC bottles flagged positive within 12 h of incubation. According to EUCAST recommendations, bacterial suspensions for DD-RAST are prepared directly from the positive bottle broth, without centrifugation or subculture, and plated promptly (within 15 min of positivity). The assay employs Mueller-Hinton agar (bioMérieux) and commercial antibiotic disks (see Table S1), with predefined zone diameter breakpoints for different incubation times. In this study, inhibition zones were read after 8 h of incubation at 35°C ± 1°C in ambient air, and susceptibility categories were interpreted using EUCAST rapid AST breakpoints for the 8 h time point.

### Reference BMD assay

Reference MIC values were determined for all bacterial isolates using the EUCAST BMD method (https://www.eucast.org/ast_of_bacteria/mic_determination) and included all 25 antimicrobial agents tested in the study, comprising 6 BL/BLI s and 19 non-BL/BLI agents (Table S1). Of these, four agents—ampicillin/sulbactam, ceftriaxone, eravacycline, and imipenem/relebactam—were excluded from the VITEK REVEAL assay; piperacillin was excluded from the VITEK 2-RAST assay; and seven agents—ampicillin/sulbactam, aztreonam, ceftriaxone, eravacycline, ertapenem, piperacillin, and tigecycline—were excluded from the DD-RAST assay.

The BMD procedure was conducted as previously described (19), following the ISO 20776-1:2019 guidelines (24), employing cation-adjusted Mueller-Hinton (caMH) broth (Thermo Fisher Scientific), and using BMD plates that were freshly, manually prepared in-house for each batch of testing. In brief, three to five colonies from each bacterial isolate were suspended in sterile saline (bioMérieux) to prepare a 0.5 McFarland standard. The resulting suspension was then diluted in caMH broth to achieve a bacterial inoculum of approximately 5 × 10^5^ CFU/mL. A 50 µL volume of this suspension was dispensed into each well of pre-prepared BMD plates containing the selected antimicrobial agents, with one plate used per isolate. Plates were incubated at 35°C in a 5% CO_2_ atmosphere and read visually to determine MIC values for all agents tested.

### Population analysis profile assay

The PAP assay was employed to investigate the presence of heteroresistant subpopulations by plating serial dilutions of a bacterial suspension onto Mueller-Hinton agar (MHA) plates containing increasing concentrations of the tested antimicrobial agent, as previously described (25, 26). For each isolate, a 10-fold dilution series (from 10^8^ to 10^2^ CFU/mL) was prepared in phosphate-buffered saline. Aliquots (100 µL) from each dilution were spread in duplicate onto MHA plates supplemented with 0, 1, 2, 4, 8, and 16 µg/mL of the antimicrobial agent. To reduce the likelihood of detecting pre-existing resistant mutants and to minimize inoculum-dependent effects, low starting cell densities were used and further diluted when necessary. After incubation at 35 °C for 24 h, colony counts were performed at each antibiotic concentration, and bacterial growth was expressed as log_10_ CFU/mL. PAP curves were then generated by plotting colony counts against antimicrobial concentrations, allowing for graphical identification of heteroresistance patterns.

### Data analysis

MIC values—or, in the case of DD-RAST, zone diameter values—were interpreted according to the 2024 EUCAST clinical breakpoints (v14.0) or, where applicable, RAST-specific breakpoints (v7.0) (27, 28). In accordance with ISO 20776-2:2021 guidelines (29), the MIC was defined as the lowest concentration of an antimicrobial agent that visibly inhibited bacterial growth after the specified incubation period. The BMD assay served as the reference method to evaluate the performance of the three RAST assays: VITEK REVEAL, VITEK 2-RAST, and DD-RAST.

Essential agreement (EA) was assessed only for VITEK REVEAL and VITEK 2-RAST and was defined as the percentage of MIC results within ±1 twofold dilution of the reference BMD MIC. Off-scale MIC values were adjusted to the nearest reportable concentration within the test range to allow inclusion in EA calculations. An EA rate ≥90% was considered acceptable (30).

Bias was calculated for VITEK REVEAL and VITEK 2-RAST, in accordance with ISO 20776-2:2021. For each antimicrobial agent with at least 25 on-scale MIC values, isolates were grouped according to their BMD MICs. The percentage of test MICs above and below the reference MIC was determined, and the difference between these percentages was used to estimate bias. Acceptable bias was defined as ranging from −30% to +30% (29).

Categorical agreement (CA) was calculated for all three RAST assays and defined as the percentage of isolates for which the test assay and the BMD assay yielded the same categorical interpretation—susceptible (S); susceptible, increased exposure (I; formerly intermediate), or resistant (R). Categorical errors were classified as very major errors (VME; false susceptible), major errors (ME; false resistant), and minor errors (mE; discrepancies between S and I or between I and R), according to ISO 20776-2:2007 criteria. A CA rate ≥90% and a VME rate <3% were considered acceptable (30).

TTR was recorded in hours and calculated for each bacterial species/antimicrobial agent combination by summing the hands-on time and the incubation time required to obtain a final result. For each assay, the mean TTR ± standard deviation (SD) was computed and stratified by susceptibility category (S, I, or R). Statistical comparisons between groups (e.g., S vs R) were performed using one-way analysis of variance or Student’s *t*-test, as appropriate. Data analysis and visualization were performed using R software (v2023.09.1) and GraphPad Prism (v10).

## RESULTS

### Overview of samples and assay scope

A total of 220 clinical GN-PBC samples were included in the study (Fig. 1), each yielding a single organism. All samples were tested using the VITEK 2-RAST assay, while subsets were also tested with the VITEK REVEAL assay (*n* = 200) and the DD-RAST assay (*n* = 164). Sample selection for each assay depended on bacterial species/antimicrobial agent compatibility. Among the 25 antibiotics tested (Table S1), 7 were BL/BLI antibiotics, including 4 next-generation antimicrobial agents: ceftazidime/avibactam, ceftolozane/avibactam, imipenem/relebactam, and meropenem/vaborbactam. Of the 220 organisms, 179 were *Enterobacterales*, 22 were *A*. *baumannii*, and 14 were *P*. *aeruginosa*. Additional species, tested only with the VITEK 2-RAST assay, included *Stenotrophomonas maltophilia* (*n* = 3), *Aeromonas caviae* (*n* = 1), and *Acinetobacter lwoffii* (*n* = 1), for a total of 18 species. As detailed in Table S2, not all organism/antibiotic combinations were tested with all three assays. However, for the four species evaluated by each assay (*E. coli*, *K. pneumoniae*, *P. aeruginosa*, and *A. baumannii*), comparative testing was performed with up to 15 antibiotics. Among these, amikacin, ciprofloxacin, imipenem, meropenem, and tobramycin were included in all three assays.

### VITEK REVEAL vs reference BMD comparison

Of the 3,614 results obtained from testing 200 organisms with the VITEK REVEAL assay, 3,603 were evaluable for performance assessment: 3,205 for 164 *Enterobacterales*, 286 for 22 *P*. *aeruginosa*, and 112 for 14 *A*. *baumannii* (Table 1). Eleven results were excluded because of MIC values falling within the area of technical uncertainty (ATU) in 10 cases, or due to a missing MIC value in the remaining case. The overall EA rate was 97.1% (3,499/3,603), the overall bias was −7.7 (307 on-scale results), and the overall CA rate was 98.3% (3,542/3,603). When stratified by organism group, EA rates were 97.1% for *Enterobacterales* (3,113/3,205), 95.8% for *P. aeruginosa* (274/286), and 100% for *A. baumannii* (112/112). Bias values were −6.9 for *Enterobacterales* (232 on-scale results) and −22.5 for *P. aeruginosa* (73 on-scale results); bias was not applicable to *A. baumannii*. The lowest EA rates (<90%) were observed for *Enterobacterales*/ceftazidime (87.1%, 142/163 results; bias: −25.0, 49 on-scale results), *P. aeruginosa*/cefepime (81.8%, 18/22 results), and *P. aeruginosa*/ceftazidime (86.4%, 19/22 results). The CA rates were 98.3% for *Enterobacterales* (3,150/3,205), 97.9% for *P. aeruginosa* (280/286), and 100% for *A. baumannii* (112/112).

**TABLE 1:** Performance of the VITEK REVEAL assay compared to the reference BMD assay for 200 Gram-negative bacterial organisms from positive blood cultures

	No. of results by the reference BMD assay^*a*^					Parameters for performance evaluation^*b*^					
	Total	For EA/CA calculation	S	I	R	EA (%; no. of results)	Bias (%; no. of on-scale results)	CA (%; no. of results)	ME (%; no. of results)	VME (%; no. of results)	mE (%; no. of results)
*Enterobacterales* organisms (*n* = 164) tested against^*c*^											
Amikacin	164	164	146	–	18	97.6; 160	–	95.7; 157	4.8; 7	0; 0	–
Amoxicillin/clavulanic acid	156	156	84	–	72	91.0; 142	–	95.6; 149	1.2; 1	8.3; 6	–
Ampicillin	90	90	35	–	55	100; 90	–	100; 90	0; 0	0; 0	–
Aztreonam	148	148	85	3	60	98.0; 145	–	97.3; 144	0; 0	0; 0	2.7; 4
Cefepime	161	161	101	3	57	90.7; 146	+6.3; 41	95.6; 154	0; 0	0; 0	4.3; 7
Cefotaxime	164	164	94	0	70	97.6; 160	–	98.8; 162	1.1; 1	0; 0	0.6; 1
Ceftazidime	164	163	93	3	67	87.1; 142	−25.0; 49	96.9; 158	0; 0	0; 0	3.1; 5
Ceftazidime/avibactam	164	164	158	–	6	96.3; 158	–	100; 164	0; 0	0; 0	–
Ceftolozane/tazobactam	143	143	114	–	29	99.3; 142	–	99.3; 142	0.9; 1	0; 0	–
Ciprofloxacin	164	156	87	0	69	98.7; 154	–	100; 156	0; 0	0; 0	–
Ertapenem	164	164	138	–	26	98.8; 162	–	99.4; 163	0.7; 1	0; 0	–
Gentamicin	164	164	127	–	37	98.2; 161	–	98.2; 161	2.4; 3	0; 0	–
Imipenem	148	148	126	0	22	97.3; 144	–	99.3; 147	0; 0	0; 0	0.7; 1
Levofloxacin	164	164	95	3	66	98.2; 161	–	96.3; 158	0; 0	0; 0	3.7; 6
Meropenem	164	164	141	3	20	98.2; 161	–	98.8; 162	0; 0	0; 0	1.2; 2
Meropenem/vaborbactam	164	164	159	–	5	99.4; 163	–	100; 164	0; 0	0; 0	–
Piperacillin	161	161	70	–	91	100; 161	–	100; 161	0; 0	0; 0	–
Piperacillin/tazobactam	164	162	126	–	36	98.8; 160	–	98.8; 160	0; 0	5.6; 2	–
Tigecycline	77	77	77	–	0	100; 77	–	100; 77	0; 0	–	–
Tobramycin	164	164	113	–	51	99.4; 163	–	98.8; 162	0.9; 1	2.0; 1	–
Trimethoprim/sulfamethoxazole	164	164	103	1	60	98.2; 161	–	96.9; 159	1.9; 2	1.7; 1	1.2; 2
Total antibiotics	3,216	3,205	2,272	16	917	97.1; 3,113	−6.9; 232	98.3; 3,150	0.7; 17	1.1; 10	2.2; 28
*Pseudomonas aeruginosa* organisms (*n* = 22) tested against											
Amikacin	22	22	21	–	1	95.5; 21	–	100; 22	0; 0	0; 0	–
Aztreonam	22	22	–	21	1	90.9; 20	–	95.5; 21	0; 0	100; 1	–
Cefepime	22	22	–	21	1	81.8; 18	–	90.9; 20	9.5; 2	0; 0	–
Ceftazidime	22	22	–	20	2	86.4; 19	–	95.5; 21	5.0; 1	0; 0	–
Ceftazidime/avibactam	22	22	21	–	1	100; 22	–	100; 22	0; 0	0; 0	–
Ceftolozane/tazobactam	22	22	21	–	1	100; 22	–	100; 22	0; 0	0; 0	–
Ciprofloxacin	22	22	–	19	3	100; 22	–	100; 22	0; 0	0; 0	–
Imipenem	22	22	–	21	1	100; 22	–	100; 22	0; 0	0; 0	–
Meropenem	22	22	20	1	1	90.9; 20	–	95.5; 21	0; 0	0; 0	4.5; 1
Meropenem/vaborbactam	22	22	22	–	0	100; 22	–	95.5; 21	4.5; 1	–	–
Piperacillin	22	22	–	20	2	100; 22	–	100; 22	0; 0	0; 0	–
Piperacillin/tazobactam	22	22	–	21	1	100; 22	–	100; 22	0; 0	0; 0	–
Tobramycin	22	22	21	–	1	100; 22	–	100; 22	0; 0	0; 0	–
Total antibiotics	286	286	126	144	16	95.8; 274	−22.5; 73	97.9; 280	1.5; 4	6.2; 1	4.5; 1
*Acinetobacter baumannii* organisms (*n* = 14) tested against											
Amikacin	14	14	2	–	12	100; 14	–	100; 14	0; 0	0; 0	–
Ciprofloxacin	14	14	–	1	13	100; 14	–	100; 14	0; 0	0; 0	–
Gentamicin	14	14	1	–	13	100; 14	–	100; 14	0; 0	0; 0	–
Imipenem	14	14	1	0	13	100; 14	–	100; 14	0; 0	0; 0	0; 0
Levofloxacin	14	14	1	0	13	100; 14	–	100; 14	0; 0	0; 0	0; 0
Meropenem	14	14	1	0	13	100; 14	–	100; 14	0; 0	0; 0	0; 0
Tobramycin	14	14	1	–	13	100; 14	–	100; 14	0; 0	0; 0	–
Trimethoprim/sulfamethoxazole	14	14	1	0	13	100; 14	–	100; 14	0; 0	0; 0	0; 0
Total antibiotics	112	112	8	1	103	100; 112	–	100; 112	0; 0	0; 0	0; 0
All organisms tested (*n* = 200)	3,614	3,603	2,406	161	1,036	97.1; 3,499	−7.7; 307	98.3; 3,542	0.8; 21	1.1; 11	2.1; 29

^*a*^ Of the total number of results obtained with the reference BMD assay for each antibiotic tested, some were excluded from the EA/CA and error rate calculations due to missing MIC values (i.e., one case in which ceftazidime was tested against *Citrobacter koseri*) or because MICs fell within the ATU. The latter included ciprofloxacin MIC values for *K. pneumoniae* (*n* = 5), *E. coli* (*n* = 2), and *Klebsiella aerogenes* (*n* = 1) isolates and piperacillin/tazobactam MIC values for *E. coli* (*n* = 1) and *K. aerogenes* (*n* = 1) isolates. Based on reference MIC values interpreted according to EUCAST breakpoints, isolates were classified as susceptible (S), susceptible, increased exposure (I), or resistant (R). The symbol “–” indicates not applicable.

^*b*^ To evaluate the performance of the VITEK REVEAL assay, key parameters, such as EA and bias, were assessed in accordance with ISO 20776-2:2021. EA was defined as MIC values within ±1 log_2_ dilution of the reference MIC, while bias was calculated for antibiotics with ≥25 on-scale MIC results, with an acceptable range of −30% to +30%. Additional parameters included CA and associated error rates—very major errors (VMEs), major errors (MEs), and minor errors (mEs).

^*c*^ Among *Enterobacterales*, the number of antibiotics tested varied by species, as organisms with known intrinsic resistance to specific antibiotics in the VITEK REVEAL assay, or for which no EUCAST breakpoints are available, were excluded from testing/analysis.

**Fig 2:**
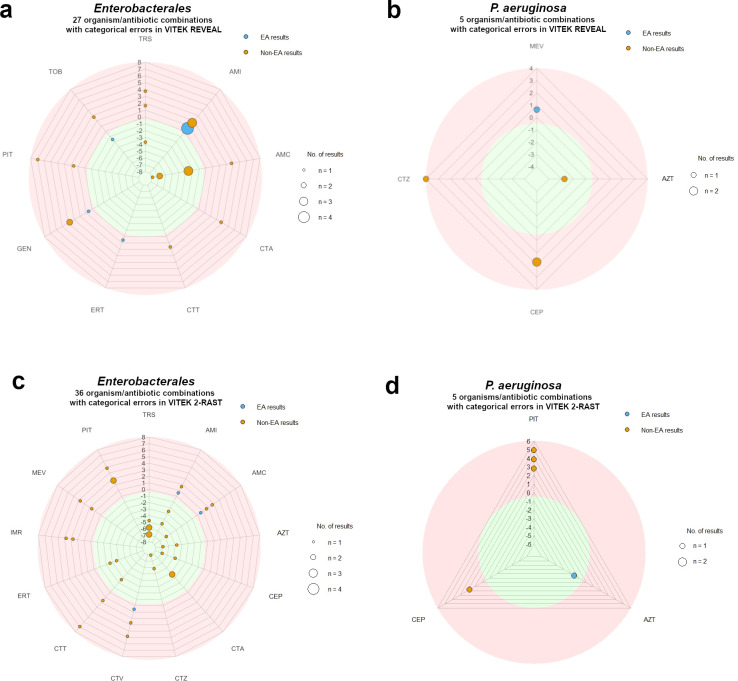
Graphical presentation of MIC results obtained by the VITEK REVEAL (**a and b**) and VITEK 2-RAST (**c and d**) assays for *Enterobacterales* (**a, c**) and *P. aeruginosa* (**b, d**) isolates showing categorical errors, stratified as either in essential agreement (EA; blue dots) or not in essential agreement (non-EA; orange dots) with reference BMD MIC results. Only major errors and very major errors were included in this analysis. Each dot represents one to four individual results, as indicated by the circle size. The radial axes represent the antibiotics tested. The shaded circular zones indicate log_2_ fold differences compared to the BMD reference: green for lower MIC values, pink for higher MIC values. The outer zones represent log_2_ differences ranging from ±1 to ±8. MIC values within ±1 log_2_ dilution from the BMD reference are considered in EA. Distances between concentric lines are for orientation only; intermediate positions are not quantitatively significant and should be interpreted as nearest integer steps. AMI, amikacin; AMC, amoxicillin/clavulanic acid; AZT, aztreonam; CEP, cefepime; CTA, cefotaxime; CTZ, ceftazidime; CTV, ceftazidime/avibactam; CTT, ceftolozane/tazobactam; ERT, ertapenem; GEN, gentamicin; IMI, imipenem; IMR, imipenem/relebactam; MER, meropenem; MEV, meropenem/vaborbactam; PIT, piperacillin/tazobactam; TOB, tobramycin; TRS, trimethoprim/sulfamethoxazole.

Among the *Enterobacterales* and *P. aeruginosa* isolates showing categorical errors with the VITEK REVEAL assay (see below for details), the corresponding organism/antibiotic combinations included MIC values that were either in EA or not in EA with the reference BMD MIC values. Figure 2a and b illustrate the distribution and direction of MIC deviations for the various organism/antibiotic combinations, emphasizing the proportion and magnitude of non-EA results and their contribution to the observed bias. Most non-EA results clustered in the pink circular area, which includes MIC values that were higher than the corresponding BMD values, with deviations ranging from +1 to +8 log_2_ dilutions. The overall upward shift of these deviations suggests a systematic MIC overestimation for certain antibiotics or organisms.

### VITEK 2-RAST vs reference BMD comparison

Of the 3,960 results obtained from testing 220 organisms with the VITEK 2-RAST assay, 3,941 were evaluable for performance assessment: 3,595 for 179 *Enterobacterales*, 264 for 22 *P*. *aeruginosa*, 70 for 14 *A*. *baumannii*, and 12 for 5 other species (Table 2). Nineteen results were excluded because the MIC values fell within the ATU. The overall EA rate was 96.2% (3,793/3,941), the overall bias was −10.4 (758 on-scale results), and the overall CA rate was 98.4% (3,876/3,941). When stratified by organism group, EA rates were 96.4% for *Enterobacterales* (3,466/3,595), 92.8% for *P. aeruginosa* (245/264), 100% for *A. baumannii* (70/70), and 100% for other species (12/12). Bias values were −12.7 for *Enterobacterales* (548 on-scale results) and +5.8 for *P. aeruginosa* (208 on-scale results); bias was not applicable to *A. baumannii* or other species. The lowest EA rates (<90%) were observed for *Enterobacterales*/cefepime (87.8%, 144/164 results; bias: −27.1, 32 on-scale results), *Enterobacterales*/ceftazidime (88.3%, 158/179 results; bias: −36.2, 53 on-scale results), *P. aeruginosa*/cefepime (72.7%, 16/22 results), and *P. aeruginosa*/piperacillin/tazobactam (77.3%, 17/22 results). The CA rates were 98.4% for *Enterobacterales* (3,537/3,595), 97.3% for *P. aeruginosa* (257/264), 100% for *A. baumannii* (70/70), and 100% for other species (12/12).

**TABLE 2:** Performance of the VITEK 2-RAST assay compared to the reference BMD assay for 220 Gram-negative bacterial organisms from positive blood cultures

	No. of results by the reference BMD assay^*a*^					Parameters for performance evaluation^*b*^					
	Total	For EA/CA calculation	S	I	R	EA (%; no. of results)	Bias (%; no. of on-scale results)	CA (%; no. of results)	ME (%; no. of results)	VME (%; no. of results)	mE (%; no. of results)
*Enterobacterales* organisms (*n* = 179) tested against^*c*^											
Amikacin	155	155	133	–	22	95.5; 148	−5.6; 109	97.4; 151	1.5; 2	9.1; 2	–
Amoxicillin/clavulanic acid	169	169	87	–	82	93.5; 158	−7.7; 34	97.6; 165	3.4; 3	1.2; 1	–
Ampicillin	156	156	34	–	122	100; 156	–	100; 156	0; 0	0; 0	–
Ampicillin/sulbactam	158	158	70	–	88	99.4; 157	−0.6; 33	100; 158	0; 0	0; 0	–
Aztreonam	179	179	114	5	60	90.5; 162	−33.2; 25	95.5; 171	0; 0	3.3; 2	3.4; 6
Cefepime	164	164	104	3	57	87.8; 144	−27.1; 32	94.5; 155	0; 0	3.5; 2	4.3; 7
Cefotaxime	164	164	94	0	70	97.0; 159	–	98.8; 162	0; 0	2.9; 2	0; 0
Ceftazidime	179	179	109	3	67	88.3; 158	−36.2; 53	97.2; 174	0; 0	3.0; 2	1.7; 3
Ceftazidime/avibactam	179	179	173	–	6	95.0; 170	−22.8; 51	98.3; 176	1.7; 3	0; 0	–
Ceftolozane/tazobactam	164	164	133	–	31	95.7; 157	−16.6; 33	98.2; 161	1.5; 2	3.2; 1	–
Ceftriaxone	164	164	97	0	67	100; 163	–	100; 163	0; 0	0; 0	0; 0
Ciprofloxacin	179	171	97	0	74	97.7; 167	–	100; 171	0; 0	0; 0	–
Eravacyclin	74	74	74	–	0	100; 74	–	100; 74	0; 0	–	–
Ertapenem	179	179	153	–	26	97.8; 175	–	98.9; 177	0; 0	7.7; 2	–
Gentamicin	176	176	138	–	38	100; 176	–	100; 176	0; 0	0; 0	–
Imipenem	151	151	129	0	22	99.3; 150	–	99.3; 150	0; 0	0; 0	0.7; 1
Imipenem/relebactam	151	151	146	–	5	98.7; 149	–	98.7; 149	1.4; 2	0; 0	–
Meropenem	179	179	156	3	20	99.4; 178	–	98.3; 176	0; 0	0; 0	1.7; 3
Meropenem/vaborbactam	179	179	174	–	5	96.6; 173	–	98.9; 177	1.1; 2	0; 0	–
Piperacillin/tazobactam	168	157	124	–	33	95.5; 150	–	98.1; 154	2.4; 3	0; 0	–
Tobramycin	168	168	117	–	51	100; 168	–	100; 168	0; 0	0; 0	–
Trimethoprim/sulfamethoxazole	179	179	114	1	64	96.6; 173	–	96.1; 172	0; 0	7.8; 5	1.1; 2
Total antibiotics	3,614	3,595	2,570	15	1,010	96.4; 3,466	−12.7; 548	98.4; 3,537	0.7; 17	1.9; 19	1.6; 22
*Pseudomonas aeruginosa* organisms (*n* = 22) tested against											
Amikacin	22	22	21	–	1	95.5; 21	–	100; 22	0; 0	0; 0	–
Aztreonam	22	22	–	21	1	90.9; 20	–	95.5; 21	0; 0	100; 1	–
Cefepime	22	22	–	21	1	72.7; 16	–	95.4; 21	4.8; 1	0; 0	–
Ceftazidime	22	22	–	20	2	90.9; 20	–	100; 22	0; 0	0; 0	–
Ceftazidime/avibactam	22	22	21	–	1	90.9; 20	–	100; 22	0; 0	0; 0	–
Ceftolozane/tazobactam	22	22	21	–	1	100; 2	–	100; 22	0; 0	0; 0	–
Ciprofloxacin	22	22	–	19	3	100; 22	–	100; 22	0; 0	0; 0	–
Imipenem	22	22	–	21	1	100; 22	–	95.5; 21	0; 0	0; 0	–
Imipenem/relebactam	22	22	22	–	0	100; 22	–	100; 22	0; 0	–	–
Meropenem	22	22	20	1	1	95.5; 21	–	90.9; 20	0; 0	0; 0	9.1; 2
Piperacillin/tazobactam	22	22	–	21	1	77.3; 17	–	86.4; 19	14.3; 3	0; 0	–
Tobramycin	22	22	21	–	1	100; 22	–	100; 22	0; 0	0; 0	–
Total antibiotics	264	264	126	124	14	92.8; 245	+5.8; 208	97.3; 257	1.6; 4	7.1; 1	9.1; 2
*Acinetobacter baumannii* organisms (*n* = 14) tested against											
Amikacin	14	14	2	–	12	100; 14	–	100; 14	0; 0	0; 0	–
Ciprofloxacin	14	14	–	1	13	100; 14	–	100; 14	0; 0	0; 0	–
Imipenem	14	14	1	0	13	100; 14	–	100; 14	0; 0	0; 0	0; 0
Meropenem	14	14	1	0	13	100; 14	–	100; 14	0; 0	0; 0	0; 0
Tobramycin	14	14	1	–	13	100; 14	–	100; 14	0; 0	0; 0	–
Total antibiotics	70	70	5	1	64	100; 70	–	100; 70	0; 0	0; 0	0; 0
Other species organisms (*n* = 5) tested against											
Amikacin	1	1	1	–	0	100; 1	–	100; 1	0; 0	–	–
Cefepime	1	1	1	0	0	100; 1	–	100; 1	0; 0	–	0; 0
Ciprofloxacin	2	2	1	1	0	100; 2	–	100; 2	0; 0	0; 0	0; 0
Levofloxacin	1	1	1	0	0	100; 1	–	100; 1	0; 0	–	0; 0
Imipenem	1	1	1	0	0	100; 1	–	100; 1	0; 0	–	0; 0
Meropenem	1	1	1	0	0	100; 1	–	100; 1	0; 0	–	0; 0
Trimethoprim/sulfamethoxazole	5	5	2	3	0	100; 5	–	100; 5	0; 0	–	0; 0
Total antibiotics	12	12	8	4	0	100; 12	–	100; 12	0; 0	0; 0	0; 0
All organisms tested (*n* = 220)	3,960	3,941	2,709	144	1,088	96.2; 3,793	−10.4; 758	98.4; 3,876	0.7; 21	1.8; 20	1.7; 24

^*a*^ Of the total number of results obtained with the reference BMD assay for each antibiotic tested, some were excluded from the EA/CA and error rate calculations due to MICs that fell within the ATU. This included ciprofloxacin MIC values for *E. coli* (*n* = 4), *K. pneumoniae* (*n* = 3), and *Citrobacter freundii *(*n* = 1) isolates and piperacillin/tazobactam MIC values for *E. coli* (*n* = 8), *K. pneumoniae* (*n* = 2), and *K. oxytoca* (*n* = 1) isolates. Based on reference MIC values interpreted according to EUCAST breakpoints, isolates were classified as susceptible (S), susceptible, increased exposure (I), or resistant (R). The symbol “–” indicates not applicable.

^*b*^ To evaluate the performance of the VITEK 2-RAST assay, key parameters, such as EA and bias, were assessed in accordance with ISO 20776-2:2021. EA was defined as MIC values within ±1 log_2_ dilution of the reference MIC, while bias was calculated for antibiotics with ≥25 on-scale MIC results, with an acceptable range of −30% to +30%. Additional parameters included CA and associated error rates—VMEs, MEs, and mEs.

^*c*^ Among *Enterobacterales* or other species groups, the number of antibiotics tested varied by species, as organisms with known intrinsic resistance to specific antibiotics in the VITEK 2-RAST assay, or for which no EUCAST breakpoints are available, were excluded from testing/analysis.

Among the *Enterobacterales* and *P. aeruginosa* isolates showing categorical errors with the VITEK 2-RAST assay (see below for details), the corresponding organism/antibiotic combinations included MIC values that were either in EA or not in EA with the reference BMD MIC values. Figure 2 (panels c and d) illustrates the distribution and direction of MIC deviations for the various organism/antibiotic combinations, emphasizing the proportion and magnitude of non-EA results and their contribution to the observed bias. Most non-EA results clustered in either the pink circular area, which includes MIC values that were higher than the corresponding BMD values (deviations from +1 to +8 log_2_ dilutions), or the green circular area, which includes MIC values that were lower than the corresponding BMD values (deviations from −1 to −8 log_2_ dilutions). These opposite shifts suggest a systematic MIC overestimation or underestimation for certain antibiotics or organisms.

### DD-RAST vs reference BMD assay comparison

Of the 2,520 results obtained from testing 164 organisms with the DD-RAST assay, 2,388 were evaluable for performance assessment: 2,003 for 128 *Enterobacterales* (74 *E. coli* and 54 *K*. *pneumoniae*), 276 for 22 *P*. *aeruginosa*, and 109 for 14 *A*. *baumannii* (Table 3). One hundred thirty-two results were excluded because of MIC values falling within the ATU in 127 cases or within the category I in the remaining 5 cases. The overall CA rate was 98.2% (2,345/2,388); when stratified by organism group, CA rates were 98.1% (1,965/2,003), 98.6% (272/276), and 99.1% (108/109), respectively.

**TABLE 3:** Performance of the DD-RAST assay compared to the reference BMD assay for 164 Gram-negative bacterial organisms from positive blood cultures

	No. of results by the reference BMD assay^*a*^					Parameters for performance evaluation^*b*^			
	Total	For CA calculation	S	I	R	CA (%; no. of results)	ME (%; no. of results)	VME (%; no. of results)	mE (%; no. of results)
*Enterobacterales* organisms (*n* = 128) tested against^*c*^									
Amikacin	128	117	104	–	13	97.4; 114	1.9; 2	7.7; 1	–
Amoxicillin/clavulanic acid	128	115	60	–	55	96.5; 111	1.7; 1	5.5; 3	–
Ampicillin	74	72	24	–	48	100; 72	0; 0	0; 0	–
Cefotaxime	128	125	67	–	58	100; 125	0; 0	0; 0	–
Ceftazidime	128	123	66	–	57	100; 123	0; 0	0; 0	–
Ceftazidime/avibactam	128	126	121	–	5	100; 126	0; 0	0; 0	–
Ceftolozane/tazobactam	128	121	93	–	28	97.5; 118	3.2; 3	0; 0	–
Ciprofloxacin	128	122	58	–	64	98.3; 120	0; 0	3.1; 2	–
Gentamicin	128	121	90	–	31	99.2; 120	1.1; 1	0; 0	–
Imipenem	128	128	106	–	22	100; 128	0; 0	0; 0	–
Imipenem/relebactam	128	127	122	–	5	100; 127	0; 0	0; 0	–
Levofloxacin	128	122	63	–	59	99.2; 121	1.6; 1	0; 0	–
Meropenem	128	125	106	–	19	100; 125	0; 0	0; 0	–
Meropenem/vaborbactam	128	126	122	–	4	94.4; 119	4.9; 6	25.0; 1	–
Piperacillin/tazobactam	128	96	63	–	33	90.6; 87	14.3; 9	0; 0	–
Tobramycin	128	109	66	–	43	94.5; 103	6.1; 4	4.7; 2	–
Trimethoprim/sulfamethoxazole	128	128	71	–	57	98.4; 126	0; 0	3.5; 2	–
Total antibiotics	2,122	2,003	1,402	–	601	98.1; 1,965	1.9; 27	1.8; 11	–
*Pseudomonas aeruginosa* organisms (*n* = 22) tested against									
Amikacin	22	22	21	–	1	100; 22	0; 0	0; 0	–
Cefepime	22	22	–	21	1	90.9; 20	4.8; 1	100; 1	–
Ceftazidime	22	20	–	18	2	100; 20	0; 0	0; 0	–
Ceftazidime/avibactam	22	20	19	–	1	100; 20	0; 0	0; 0	–
Ceftolozane/tazobactam	22	22	21	–	1	100; 22	0; 0	0; 0	–
Ciprofloxacin	22	20	–	18	2	95.0; 19	0; 0	50.0; 1	–
Levofloxacin	22	22	–	20	2	100; 22	0; 0	0; 0	–
Imipenem	22	22	–	20	2	100; 22	0; 0	0; 0	–
Imipenem/relebactam	22	22	22	–	0	100; 22	0; 0	0; 0	–
Meropenem	22	21	20	–	1	95.2; 20	5.0; 1	0; 0	–
Meropenem/vaborbactam	22	22	22	–	0	100; 22	0; 0	–	–
Piperacillin/tazobactam	22	19	–	18	1	100; 19	0; 0	0; 0	–
Tobramycin	22	22	21	–	1	100; 22	0; 0	0; 0	–
Total antibiotics	286	276	146	115	15	98.6; 272	0.8; 2	13.3; 2	–
*Acinetobacter baumannii* organisms (*n* = 14) tested against									
Amikacin	14	13	2	–	11	100; 13	0; 0	0; 0	–
Ciprofloxacin	14	14	–	1	13	100; 14	0; 0	0; 0	–
Gentamicin	14	13	1	–	12	100; 13	0; 0	0; 0	–
Imipenem	14	14	1	–	13	100; 14	0; 0	0; 0	–
Levofloxacin	14	14	1	–	13	92.9; 13	100; 1	0; 0	–
Meropenem	14	14	1	–	13	100; 14	0; 0	0; 0	–
Tobramycin	14	13	1	–	12	100; 13	0; 0	0; 0	–
Trimethoprim/sulfamethoxazole	14	14	1	–	13	100; 14	0; 0	0; 0	–
Total antibiotics	112	109	8	1	100	99.1; 108	11.1; 1	0; 0	–
All organisms tested (*n* = 164)	2,520	2,388	1,556	116	716	98.2; 2,345	1.8; 30	1.8; 13	–

^*a*^ Of the total number of results obtained with the reference BMD assay for each antibiotic tested, some were excluded from the CA and error rate calculations due to inhibition zone diameters falling within the ATU. This included the results for amikacin tested against *E. coli* (*n* = 10), *K. pneumoniae* (*n* = 1), and *A. baumannii* (*n* = 1); amoxicillin/clavulanic acid against *K. pneumoniae* (*n* = 8) and *E. coli* (*n* = 6); ampicillin against *E. coli* (*n* = 2); cefotaxime against *K. pneumoniae* (*n* = 2) and *E. coli* (*n* = 1); ceftazidime against *K. pneumoniae* (*n* = 2), *P. aeruginosa* (*n* = 2), and *E. coli* (*n* = 1); ceftazidime/avibactam against *K. pneumoniae* (*n* = 2) and *P. aeruginosa* (*n* = 2); ceftolozane/tazobactam against *K. pneumoniae* (*n* = 4) and *E. coli* (*n* = 3); ciprofloxacin against *K. pneumoniae* (*n* = 4), *E. coli* (*n* = 3), and *P. aeruginosa* (*n* = 2); gentamicin against *E. coli* (*n* = 5), *K. pneumoniae* (*n* = 2), and *A. baumannii* (*n* = 1); levofloxacin against *E. coli* (*n* = 3) and *K. pneumoniae* (*n* = 2); meropenem against *P. aeruginosa* (*n* = 1); meropenem/vaborbactam against *K. pneumoniae* (*n* = 2); piperacillin/tazobactam against *E. coli* (*n* = 25), *K. pneumoniae* (*n* = 7), and *P. aeruginosa* (*n* = 3); and tobramycin against *E. coli* (*n* = 18), *K. pneumoniae* (*n* = 1), and *A. baumannii* (*n* = 1). Based on reference MIC values interpreted according to EUCAST breakpoints, isolates were classified as susceptible (S), susceptible, increased exposure (I), or resistant (R). Consequently, results with MIC values falling within the I category were excluded from CA and error rate calculations, as follows: ceftazidime tested against *K. pneumoniae* (*n* = 2) and *E. coli* (*n* = 1); levofloxacin against *K. pneumoniae* (*n* = 1); meropenem against *K. pneumoniae* (*n* = 1). The symbol “–” indicates not applicable.

^*b*^ To evaluate the performance of the DD-RAST assay, key parameters—such as CA and associated error rates, including VMEs and MEs—were calculated. mEs were not calculated.

^*c*^ Among *Enterobacterales* (74 *E. coli* and 54 *K. pneumoniae*), the number of antibiotics tested varied only for *K. pneumoniae*, as this species was not tested against ampicillin in the DD-RAST assay due to the lack of EUCAST breakpoints.

### Head-to-head comparison of the three study assays

By testing the 164 GN organisms with all three assays, 2,978, 3,096, and 2,520 organism/antibiotic combinations were generated with the VITEK REVEAL, VITEK 2-RAST, and DD-RAST assays, respectively, of which 2,969, 3,078, and 2,388 were evaluable for comparative performance assessment. While Tables 1–3 report individual assay performance metrics (EA and CA), Table 4 is designed to facilitate a direct, side-by-side comparison of the three assays. This comparison is applicable only to *E. coli* and *K. pneumoniae* among *Enterobacterales*. The overall EA rates were 97.5% for VITEK REVEAL (2,896/2,969) and 96.3% for VITEK 2-RAST (2,967/3,078); bias values were −7.1 (249 on-scale results) and −6.9 (624 on-scale results), respectively. The overall CA rates were 98.4% (2,921/2,969) for VITEK REVEAL, 98.4% (3,029/3,078) for VITEK 2-RAST, and 98.2% (2,345/2,388) for DD-RAST.

**TABLE 4:** Comparison of the three study assays for 164 Gram-negative bacterial organisms from positive blood cultures

	Assay name		
	VITEK REVEAL	VITEK 2-RAST	DD-RAST
Assay details			
Total organisms tested (no.)^*a*^	164	164	164
Total antibiotics tested for *E. coli* (no.)	21	22	17
Total antibiotics tested for *K. pneumoniae* (no.)	19	21	16
Total antibiotics tested for *P. aeruginosa* (no.)	13	12	13
Total antibiotics tested for *A. baumannii* (no.)	8	5	8
Total organism/antibiotic combinations evaluated/tested (no.)^*b*^	2,969/2,978	3,078/3,096	2,388/2,520
S organism/antibiotic combinations (no.)^*c*^	1,876	1,980	1,556
I organism/antibiotic combinations (no.)^*d*^	155	134	116
R organism/antibiotic combinations (no.)^*e*^	938	964	716
Assay performance^*f*^			
Essential agreement (%; no.)	97.5; 2,896	96.3; 2,967	–
Bias (%; no.)	−7.1; 249	−6.9; 624	–
Categorical agreement (%; no.)	98.4; 2,921	98.4; 3,029	98.2; 2,345
Major errors (%; no.)	0.9; 18	1.0; 21	1.8; 30
Very major errors (%; no.)	1.2; 11	1.2; 11	1.8; 13
Minor errors (%; no.)	2.0; 19	2.2; 17	–

^*a*^ All 164 organisms presented here belong to *E. coli* (*n* = 74), *K. pneumoniae* (*n* = 54), *P. aeruginosa* (*n* = 22), and *A. baumannii* (*n* = 14)—the only Gram-negative species for which the EUCAST disk diffusion (DD)-based RAST method is currently applicable (https://www.eucast.org/rapid_ast_in_bloodcultures).

^*b*^ Of the total of organism/antibiotic combinations tested, 4,440 involved *E. coli* (1,554 with VITEK REVEAL; 1,628 with VITEK 2-RAST; 1,258 with DD-RAST), 3,024 involved *K. pneumoniae* (1,026 with VITEK REVEAL; 1,134 with VITEK 2-RAST; 864 with DD-RAST), 836 involved *P. aeruginosa* (286 with VITEK REVEAL; 264 with VITEK 2-RAST; 286 with DD-RAST), and 294 involved *A. baumannii* (112 with VITEK REVEAL; 70 with VITEK 2-RAST; 112 with DD-RAST). The numerators in each ratio indicate the number of organism/antibiotic combinations used for EA (excluding DD-RAST) and CA calculations (see also Tables 1–3).

^*c*^ S = susceptible, based on EUCAST breakpoints.

^*d*^ I = susceptible, increased exposure, based on EUCAST breakpoints.

^*e*^ R = resistant, based on EUCAST breakpoints.

^*f*^ The symbol “–” indicates not applicable.

### Analysis of discordant results

Categorical errors observed with the three assays are summarized in Table 5. Among the 18 GN bacterial species tested, nine exhibited at least one categorical error. The antibiotics most frequently associated with ME, VME, and mE were piperacillin/tazobactam (*n* = 15), amoxicillin/clavulanic acid (*n* = 10), and cefepime (*n* = 14), respectively. Excluding *A. caviae*, *A. lwoffii*, and *S. maltophilia*—which were tested only with VITEK 2-RAST and showed no errors—categorical discrepancies involved a variety of organism/antibiotic combinations across the three assays (Tables 1–3). Of the total errors with the VITEK REVEAL (*n* = 61) or VITEK 2-RAST (*n* = 65), 54 (88.5%) and 50 (76.9%), respectively, had an MIC value bracketing the breakpoint (within ±1 twofold dilution). Of the total errors with the DD-RAST (*n* = 43), 31 (72.1%) had a zone diameter value bracketing the breakpoint (within 3 mm).

**TABLE 5:** Categorical errors observed with Gram-negative bacterial organisms from positive blood cultures tested by the three study assays

Bacterial species^*a*^	Type of errors for organism/antibiotic combinations (no.) by assay^*b*^							
	VITEK REVEAL			VITEK 2-RAST			DD-RAST	
	ME	VME	mE	ME	VME	mE	ME	VME
*A. baumannii*							LEV (1)	
*C. freundii*			AZT (1)	PIT (1)				
			CEP (1)					
			LEV (1)					
*E. cloacae* complex					CTT (1)			
					ERT (1)			
*E. coli*	AMI (7)	AMC (6)	AZT (1)	AMI (2)	AMI (2)	AZT (5)	AMI (2)	AMI (1)
	GEN (2)	PIT (2)	CEP (2)	AMC (3)	AMC (1)	CEP (3)	AMC (1)	AMC (2)
	TRS (1)	TRS (1)	CTA (1)	CTT (1)	AZT (2)	CTZ (2)	CTT (2)	CIP (1)
			CTZ (3)	PIT (1)	CEP (1)		GEN (1)	TRS (1)
			LEV (2)		CTZ (2)		PIT (7)	
					TRS (2)		TOB (4)	
*K. aerogenes*	CTT (1)		AZT (1)			CTZ (1)		
*K. oxytoca*			CEP (1)			CEP (1)		
*K. pneumoniae*	AMC (1)	TOB (1)	AZT (1)	CTV (3)	CTA (1)	AZT (1)	CTT (1)	AMC (1)
	CTA (1)		CTZ (2)	CTT (1)	ERT (1)	IMI (1)	LEV (1)	CIP (1)
	ERT (1)		IMI (1)	IMR (2)	TRS (3)	MER (3)	MEV (6)	MEV (1)
	TRS (1)		LEV (2)	MEV (2)		TRS (1)	PIT (2)	TOB (2)
			MER (2)	PIT (1)				TRS (1)
			TRS (1)					
*P. aeruginosa*	CEP (2)	AZT (1)	MER (1)	CEP (1)	AZT (1)	MER (2)	CEP (1)	CEP (1)
	CTZ (1)			PIT (3)			MER (1)	CIP (1)
	MEV (1)							
*P. mirabilis*	GEN (1)		CEP (3)		CEP (1)	CEP (3)		
	TOB (1)		LEV (1)		CTA (1)	TRS (1)		
			TRS (1)					

^*a*^ Nine of the 18 bacterial species included in the study showed categorical errors when the corresponding organisms were tested with the VITEK REVEAL, VITEK 2-RAST, and DD-RAST assays.

^*b*^ Errors were calculated upon categorization of bacterial organisms as S (susceptible), I (susceptible, increased exposure), or R (resistant) based on the reference MIC results obtained for each organism/antibiotic combination studied, and classified as VME, ME, or mE. Errors in the last category were not calculated for the DD-RAST assay. Numbers in parentheses indicate the organisms with the specified type of error, which occurred when testing them against the indicated antibiotics. AMI, amikacin; AMC, amoxicillin/clavulanic acid; AZT, aztreonam; CEP, cefepime; CIP, ciprofloxacin; CTA, cefotaxime; CTT, ceftolozane/tazobactam; CTV, ceftazidime/avibactam; CTZ, ceftazidime; ERT, ertapenem; GEN, gentamicin; IMI, imipenem; IMR, imipenem/relebactam; LEV, levofloxacin; MER, meropenem; MEV, meropenem/vaborbactam; PIT, piperacillin/tazobactam; TOB, tobramycin; TRS, trimethoprim/sulfamethoxazole.

Focusing on organisms tested with all three assays (*E. coli*, *K. pneumoniae*, *P. aeruginosa*, and *A. baumannii*; see Table 4), ME rates were 0.9% (*n* = 18), 1.0% (*n* = 21), and 1.8% (*n* = 30), and VME rates were 1.2% (*n* = 11), 1.2% (*n* = 11), and 1.8% (*n* = 13), for VITEK REVEAL, VITEK 2-RAST, and DD-RAST, respectively. Minor errors were observed only with VITEK REVEAL (2.0%, 19 errors) and VITEK 2-RAST (2.2%, 17 errors).

Considering only the 164 GN organisms tested with all three assays (Table 5), concordant categorical errors involving the same antibiotic were occasionally observed. Seventeen organisms exhibited errors against ten antibiotics in at least two assays, for a total of 18 errors. These included MEs (4/18, 22.2%), VMEs (4/18, 22.2%), and minor errors (10/18, 55.6%). Among these, nine occurred with both VITEK REVEAL and VITEK 2-RAST, six with both VITEK 2-RAST and DD-RAST, and three with both VITEK REVEAL and DD-RAST. In two cases—1 *P*. *aeruginosa*/cefepime ME and 1 *E. coli*/trimethoprim-sulfamethoxazole VME—the same organism/antibiotic combination was misclassified by all three assays.

**Fig 3:**
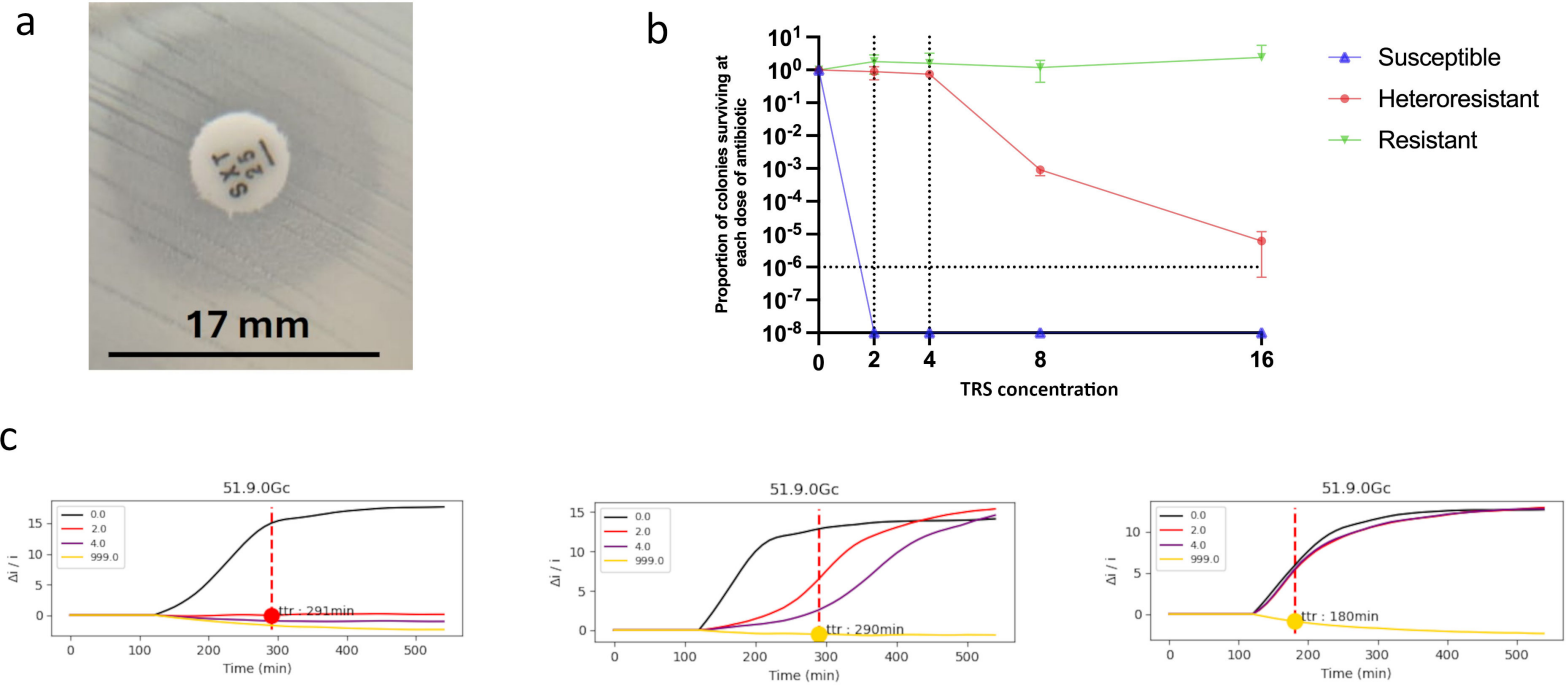
Heteroresistance assessment using the PAP assay. A *K. pneumoniae* organism, categorized as false resistant to trimethoprim/sulfamethoxazole (TRS) by the VITEK REVEAL assay (MIC > 4 µg/mL), was selected for heteroresistance analysis. The corresponding isolate—recovered in culture—was subjected to the PAP assay due to a double inhibition zone observed on disk diffusion testing: an outer zone (17  mm) consistent with susceptibility and an inner zone suggestive of a resistant subpopulation (panel **a**). The organism was also categorized as susceptible by the VITEK 2-RAST assay (MIC ≤ 20 µg/mL). PAP curves of the test isolate and representative TRS-susceptible and TRS-resistant control isolates are shown in panel **b**. The proportion of CFU recovered from the starting inoculum (10^1^ to 10^-8^; y-axis) is plotted against increasing twofold TRS concentrations (0 to 16 µg/mL; x-axis). The test isolate was defined as heteroresistant based on its ability to grow at TRS concentrations at least twofold higher than the susceptibility breakpoint. Panel **c** shows the VOC-based growth curves automatically obtained with the VITEK REVEAL assay for the three organisms (TRS-susceptible, test organism, and TRS-resistant) at the time of direct testing. These original curves were subsequently reviewed after performing the PAP assay on the corresponding isolates. In each graph, growth kinetics are shown in the absence (black curve) and presence (red and pink curves) of the antibiotic. In the middle graph, representing the test organism, the red and pink curves are split, unlike in the left and right graphs, where the two curves completely overlap.

Discrepancies were only described and not formally investigated. However, a preliminary attempt to explore potential underlying causes was performed using the PAP assay, with findings for one representative case shown in Fig. 3. Isolates were selected for PAP testing based on real-time phenotypic features suggestive of heteroresistance, such as the appearance of colonies within the inhibition zone on disk diffusion, a pattern previously proposed as indicative of this phenotype (31). Only one discrepant isolate—*K*. *pneumoniae* falsely categorized as resistant to trimethoprim/sulfamethoxazole by the VITEK REVEAL assay—fulfilled these predefined selection criteria (Fig. 3a). The isolate demonstrated a heteroresistant profile, with a subpopulation surviving antibiotic concentrations at least twofold above the clinical breakpoint (Fig. 3b). Although no other discrepant cases exhibited similar real-time clues at the time of testing, retrospective review of VITEK REVEAL output identified comparable growth behaviors—specifically, divergence between the two antibiotic-exposed curves (Fig. 3c)—in a few additional isolate/antibiotic combinations (i.e., *E. coli*/gentamicin, *P. mirabilis*/tobramycin, and *P. aeruginosa*/cefepime). These three isolates were subsequently recovered and tested by PAP; however, no heteroresistant subpopulations were detected, in line with the known instability of this phenotype, which can be lost upon subculturing (32). Taken together, these observations highlight the potential value of incorporating early phenotypic signals to guide further heteroresistance testing and the need for cautious interpretation of resistance calls in rapid AST results.

### TTR comparison across the three study assays

**Fig 4:**
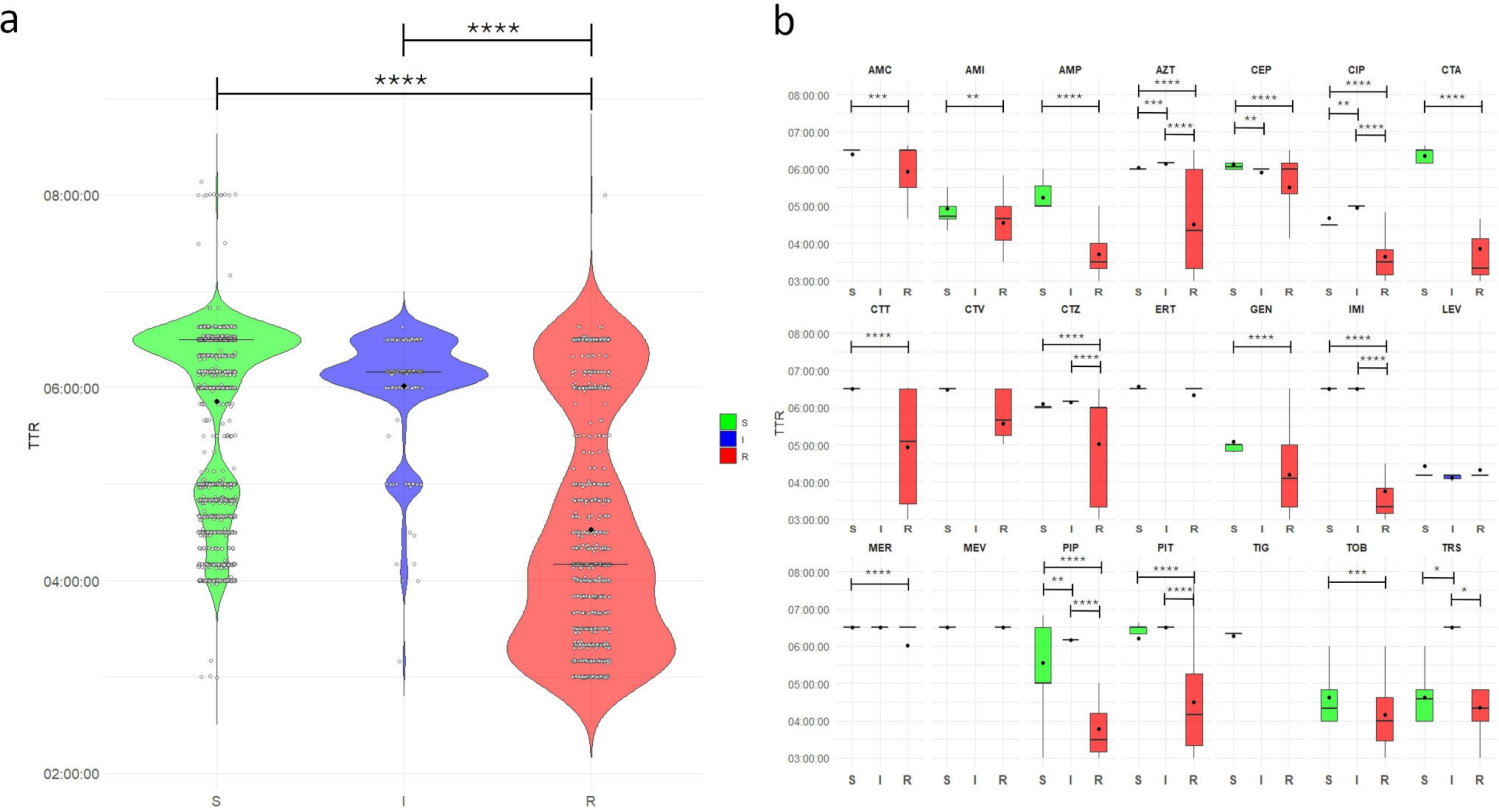
Distribution of TTR values obtained with the VITEK REVEAL assay. (**a**) Violin plot showing the distribution of TTR values for 3,603 antimicrobial agent/bacterial species combinations, stratified by susceptibility category (S, I, R). Each dot represents a TTR value for a single antimicrobial agent tested against a specific bacterial species. Thick horizontal lines indicate the median TTR; violin width reflects data density. (**b**) Box plots showing the distribution of TTR values for the same data set, stratified by susceptibility category (S, I, R) and by antimicrobial agent. Boxes represent the interquartile range; whiskers indicate the full data range; black dots represent the median TTR. TTR values on the y-axis are expressed in hours. S, susceptible; I, susceptible, increased exposure; R, resistant. Datapoints around the 8-h mark in panel a correspond to the maximum runtime of the VITEK REVEAL assay. Asterisks indicate statistically significant differences between susceptibility categories (S vs R, S vs I, and I vs R) as follows: *, *P* < 0.05; **, *P* < 0.01; ***, *P* < 0.001; ****, *P* < 0.0001.

TTR values for all 220 GN bacterial organisms included in the study are summarized in Table 6 and visualized in Fig. 4. The TTR with the DD-RAST assay was fixed at 08:00 hours, whereas mean TTRs (range) with the VITEK REVEAL and VITEK 2-RAST assays were 06:32 (04:10–08:08) and 13:51 (07:45–18:49), respectively, across all tested species. Excluding bacterial species tested only with the VITEK 2-RAST assay—namely five *Enterobacterales* (*S. marcescens*, *Morganella morganii*, *Salmonella* species, *Providencia stuartii*, and *Raoultella ornithinolytica*) and three non-*Enterobacterales* species (*A. caviae*, *A. lwoffii*, and *S. maltophilia*; see also Table S2)—the species with the shortest TTRs (mean, range) in both the VITEK REVEAL and VITEK 2-RAST assays were *A. baumannii* (05:52, 04:10–06:38; and 10:34, 08:30–17:59) and *E. cloacae* complex (05:56, 04:48–06:30; and 13:41, 08:46–17:59).

**TABLE 6:** Time to result for 220 Gram-negative bacterial organisms from positive blood cultures

Bacterial species^*a*^	No. of organisms tested	Time to result, expressed as mean (range), by assay		
		VITEK REVEAL	VITEK 2-RAST	DD-RAST
*Escherichia coli*	74	06:30 (06:30–06:38)	10:54 (07:45–16:45)	08:00
*Klebsiella pneumoniae*	54	06:30 (06:30–06:38)	15:13 (08:58–18:04)	08:00
*Pseudomonas aeruginosa*	22	06:30 (06:30–06:38)	14:35 (07:51–17:59)	08:00
*Proteus mirabilis*	16	06:54 (06:30–08:08)	17:43 (14:50–18:04)	–
*Acinetobacter baumannii*	14	05:52 (04:10–06:38)	10:34 (08:30–17:59)	08:00
*Klebsiella oxytoca*	9	06:30 (06:30–06:32)	14:56 (09:54–18:00)	–
*Serratia marcescens*	8	–	11:54 (08:55–17:55)	–
*Citrobacter koseri*	3	08:00 (08:00–08:00)	13:44 (11:09–18:04)	–
*Enterobacter cloacae* complex	3	05:56 (04:48–06:30)	13:41 (08:46–17:59)	–
*Klebsiella aerogenes*	3	07:00 (06:30–08:00)	14:24 (10:11–17:30)	–
*Morganella morganii*	3	–	11:32 (09:27–12:53)	–
*Stenotrophomonas maltophilia*	3	–	18:44 (18:41–18:49)	–
*Citrobacter freundii*	2	08:00 (08:00–08:00)	11:39 (08:58–14:20)	–
*Salmonella* species	2	–	18:00 (17:59–18:02)	–
*Aeromonas caviae*	1	–	12:11 (–)	–
*Acinetobacter lwoffii*	1	–	18:00 (–)	–
*Providencia stuartii*	1	–	10:22 (–)	–
*Raoultella ornithinolytica*	1	–	11:07 (–)	–
Total species	220	06:32 (04:10–8:08)	13:51 (07:45–18:49)	08:00

^*a*^ Bacterial species identification was performed using MALDI-TOF mass spectrometry, as described in the text. The two *Salmonella* species underwent serotyping, which identified both as *Salmonella enterica* serovar Typhimurium. The symbol “–” indicates not applicable.

When analyzed by susceptibility category, TTRs were significantly shorter for R organisms compared to S or I ones (*P* < 0.0001 for both comparisons) (Fig. 4a). Stratification by an antimicrobial agent confirmed these differences, with 15 of 21 antibiotics showing significantly shorter TTRs for R than for S or I categories (*P* < 0.05 for all comparisons) (Fig. 4b). Four of the 15 antibiotics—amoxicillin/clavulanic acid, ceftazidime/avibactam, ceftolozane/tazobactam, and piperacillin/tazobactam—were BL/BLI antibiotics.

## DISCUSSION

This study compared the performance of three RAST assays—VITEK REVEAL, VITEK 2-RAST, and DD-RAST—against the reference BMD assay using a large set of clinical GN-PBC samples. When each assay was evaluated individually, EA values were comparable between VITEK REVEAL and VITEK 2-RAST (approximately 97.0%), while CA was similarly high across all three assays (approximately 98.0%). In the subset of GN-PBC samples tested with all three assays, VITEK REVEAL showed a slightly higher EA (97.5%) than VITEK 2-RAST (96.3%). CA values remained consistent among the three assays (98.4%, 98.4%, and 98.2%), with categorical VMEs well below the 3.0% acceptance criterion. TTR for VITEK REVEAL was shorter than for VITEK 2-RAST, and also lower than the fixed 8 h TTR used for DD-RAST.

When analyzing the subset of 164 samples tested with all three RAST assays, errors appeared sporadic and not assay-dependent. Rather, they seemed to stem from the specific interaction between the antimicrobial agent and the tested organism—that is, the susceptibility category assigned for a given organism/antibiotic combination was not consistently replicated across assays. This observation supports the view that, in the RAST experimental setting, the antimicrobial agent functions as the independent variable, while the organism represents the dependent one. Such inconsistencies, previously noted in the literature, reflect an intrinsic challenge of RAST and are likely attributable to the dynamic and multifactorial nature of early phenotypic growth responses (33).

In our VITEK REVEAL assay evaluation, 6 of the 11 VMEs involved amoxicillin/clavulanic acid and were limited to *Enterobacterales*. The remaining five VMEs were distributed across other antibiotics, including one VME with aztreonam in *P. aeruginosa*; no VMEs were observed in *A. baumannii*. This pattern mirrors findings from the recent study by Ostermann et al. (11), which reported that most VMEs were associated with amoxicillin/clavulanic acid (14 of 34 total errors). The authors attributed this to the absence of an intermediate category in the EUCAST breakpoints and to the clustering of MIC values around the susceptibility threshold (≤8 mg/L), where even a single log_2_ dilution shift could generate a VME. Notably, Ostermann et al. demonstrated that recalculating the VME rate while excluding one-dilution discrepancies reduced it from 51.9% to 18.5%, suggesting that many errors were due to borderline cases. In our data set, however, the discrepancies appeared to be more substantial. The six organisms displaying VME with amoxicillin/clavulanic acid included four with REVEAL MICs of 8 mg/L and reference MICs ranging from 32 to >128 mg/L, and two with REVEAL MICs of ≤4 mg/L and reference MICs of 16 and >128 mg/L, respectively.

Our evaluation of VITEK 2-RAST and DD-RAST assays with respect to VMEs led to two main observations. First, unlike the VITEK REVEAL assay—where most VMEs clustered around amoxicillin/clavulanic acid—errors in VITEK 2-RAST and DD-RAST were distributed across a broader range of antibiotics. Specifically, among *Enterobacterales*, VMEs involved nine agents with VITEK 2-RAST and six with DD-RAST. Second, in both assays, the remaining VMEs concerned *P. aeruginosa*: one case with aztreonam in VITEK 2-RAST, and two cases in DD-RAST—one with cefepime and one with ciprofloxacin. *A. baumannii* in our study represented a highly resistant subset, with 12 to 13 of the 14 isolates exhibiting resistance to at least one tested antimicrobial agent. This trend aligns with the notion—also noted by Ostermann et al. (11)—that a low proportion of resistant isolates in the data set may increase the likelihood of VMEs, particularly when using breakpoint frameworks that lack an intermediate susceptibility category, such as EUCAST.

This observation ties into another relevant aspect we explored—namely, the TTR. According to our data (Table 6), *A. baumannii* was the species for which MICs were obtained in the shortest time with the VITEK REVEAL assay and, to a lesser extent, also with VITEK 2-RAST. This finding aligns with the dynamic described by Idelevich and Becker (33), who noted that resistant isolates typically begin growing immediately or shortly after a brief lag phase in the presence of antibiotics, generating a growth curve similar to that of the antibiotic-free control. In contrast, susceptible isolates often exhibit a prolonged lag phase and reduced initial biomass. Thus, the rapid response observed for *A. baumannii*, a predominantly resistant organism group in our study, not only helps explain the absence of VMEs but also reinforces the relevance of growth kinetics in early phenotypic testing.

While the present study does not support the preferential use of one RAST assay over another based solely on performance parameters, certain clinical scenarios require rapid turnaround times (22, 34, 35). In this context, the VITEK REVEAL assay achieved a mean TTR approximately half that of the VITEK 2-RAST assay, representing a substantial time reduction that could enhance the early management of patients with GN bacteremia.

A potential biological explanation for some of the discrepancies observed with the VITEK REVEAL assay—particularly those not attributable to borderline MICs or assay performance—lies in the phenomenon of heteroresistance (32). This condition, defined by the presence of a minor resistant subpopulation within an otherwise susceptible isolate (26), is known to compromise the reliability of phenotypic AST assays (31). In a recent study, heteroresistant isolates accounted for up to 25% of AST discrepancies, compared to less than 1% among fully susceptible isolates (31). In our data set, the presence of heteroresistance was detected by PAP assay in only one case—a *K. pneumoniae* isolate falsely categorized as resistant to trimethoprim/sulfamethoxazole. Additional isolates showing comparable growth patterns in the VITEK REVEAL assay were retrospectively identified and tested, but no heteroresistant subpopulations were recovered, likely due to the inherent instability of this phenotype (36). Although limited in scope, these findings suggest that heteroresistance may contribute to at least a subset of discordant results in rapid AST and support the need for further studies to better define its role and detectability under direct testing conditions.

An integrated approach that considers the microbial species present in each PBC sample may help optimize the use of available RAST assays. Our data suggest that certain species—such as *A. baumannii* and *P. aeruginosa*—which yielded no or very few errors and are frequently associated with multidrug resistance, may particularly benefit from testing with the VITEK REVEAL assay. In contrast, other species not supported by VITEK REVEAL—such as *A. lwoffii*, *S. maltophilia*, and *A. caviae*—can be tested using the VITEK 2-RAST assay, which provides broader species coverage. Notably, these three species (five organisms in total) showed no errors in our evaluation. It is worth noting, however, that the VITEK 2-RAST protocol used in this study is neither CE-IVD certified nor FDA-cleared, unlike the VITEK REVEAL assay. Since PBC is one of the most suitable sample types for rapid testing, particular attention should be paid to the preparation of the bacterial pellet. In this regard, the VITEK REVEAL system has been optimized by the manufacturer to function with a minimal amount of direct sample, offering a potentially more standardized and streamlined workflow.

This study has both strengths and limitations. Notably, to our knowledge, this is the first study to directly compare the MIC-based VITEK REVEAL and VITEK 2-RAST systems with the categorical DD-RAST method using a standardized BMD reference, providing relevant data for laboratories considering the adoption of rapid AST methods, particularly within EUCAST settings. We evaluated the performance of the MIC-based assays VITEK REVEAL and VITEK 2-RAST using ISO 20776-2:2021-recommended metrics—EA for agreement and bias for accuracy (30). These parameters, however, were not applicable to DD-RAST, which only provides categorical S/R results based on disk diffusion breakpoints and does not yield MICs. While our three-assay comparison remains relevant—especially in the context of prior studies (10, 11, 19)—it may inherently disadvantage DD-RAST, for which only CA could be assessed. Second, the number of organisms tested for specific species or antibiotics was limited, potentially affecting the robustness of performance estimates. Furthermore, the distribution of S and R organisms varied by species, with *A. baumannii* representing a predominantly resistant group. Nevertheless, the number of resistant MIC results obtained for *Enterobacterales* (917 with REVEAL and 1,010 with VITEK 2-RAST) and *P. aeruginosa* (16 with REVEAL and 14 with VITEK 2-RAST)—all prospectively derived from GN-BSI cases—appears comparable or superior to prior data sets (9, 11). In addition, although only one isolate per patient was included to minimize overrepresentation, clonal relatedness was not formally assessed. Therefore, we cannot exclude the possibility that some isolates may have shared a common genetic background, especially in the case of resistant isolates, which may have biased the performance estimates. Third, the use of EUCAST breakpoints, which lack an intermediate category for some antimicrobials, may have contributed to the observed VME rates. Fourth, the fixed 8-h endpoint adopted for DD-RAST was based on our early experience and may not reflect the earliest reliable time point (15). Fifth, the VITEK 2-RAST protocol required manual pellet preparation, unlike the VITEK REVEAL assay, which uses a simple dilution method optimized for direct PBC testing. Sixth, ME and VME results were not retested to assess reproducibility, limiting the ability to determine whether these discrepancies were due to random error or systematic issues. Moreover, a detailed analysis of essential disagreement (non-EA) results was not conducted for each antibiotic. However, lower EA values observed for ceftazidime and cefepime in both VITEK REVEAL and VITEK 2-RAST may be partly explained by the wide MIC ranges tested for these antibiotics, which increase the likelihood of non-EA, especially near clinical breakpoints. As previously highlighted in our REVEAL verification study (19), discrepancies may have been partly due to the use of a different cefepime powder source than that used for other antibiotics during BMD plate preparation. However, in the present study, the same BMD reference method was applied across all assays, suggesting that additional factors may underlie the lower EA observed with both VITEK REVEAL and VITEK 2-RAST. Seventh, although the shorter TTR observed with VITEK REVEAL may offer clinical benefits, we did not assess its actual impact on patient management or outcomes. Moreover, this advantage should be weighed against economic considerations, including potential costs for instrument acquisition and implementation, especially given that the VITEK REVEAL assay does not run on the VITEK 2 system. Hands-on time is also a relevant consideration: in our experience, VITEK REVEAL required 3 min and 41 s (±27 s) per sample, compared to 7 min and 22 s (±15 s) for DD-RAST. These aspects were not analyzed in the present study. Eight, this single-center evaluation focused on RAST assays that were available in our laboratory during the study period (VITEK REVEAL, VITEK 2-RAST, and DD-RAST), either in routine use or under formal evaluation. Other promising systems, such as QMAC-dRAST (QuantaMatrix Inc., Seoul, Republic of Korea), recently reviewed elsewhere (6), were not available in our setting and thus not included, which may limit the generalizability of our findings. Finally, all blood cultures in this study were processed using the BacT/ALERT Virtuo system (bioMérieux), currently adopted in our laboratory following comparative evaluations with the BD Bactec FX system (37, 38). The performance of the evaluated RAST assays with positive bottles generated by other blood culture systems remains unknown.

In conclusion, VITEK REVEAL, VITEK 2-RAST, and DD-RAST are affordable, user-friendly tools capable of delivering accurate and actionable AST results. Among these, the VITEK REVEAL assay stands out for its higher degree of standardization and near-complete automation, making it particularly suitable for high-throughput clinical microbiology laboratories. Ultimately, the decision of which RAST assay to implement will also depend on how seamlessly it integrates into existing laboratory workflows—a factor that may directly impact the reduction of TTR, a clinically relevant parameter in the management of GN-BSI caused by antimicrobial-resistant organisms.

## SUPPLEMENTAL MATERIAL

Table S1 jcm.01050-25-s0001.pdf Antimicrobial agents and corresponding concentrations for the antimicrobial susceptibility testing assays studied.

Table S2 jcm.01050-25-s0002.pdf Antimicrobial agents tested against Gram-negative bacterial species in the three RAST assays evaluated.

ASM does not own the copyrights to Supplemental Material that may be linked to, or accessed through, an article. The authors have granted ASM a non-exclusive, world-wide license to publish the Supplemental Material files. Please contact the corresponding author directly for reuse.
