# Supplementary material for: Improving time-to-result: head-to-head comparison of three rapid AST systems for Gram-negative bacteremia, including the newly developed VITEK REVEAL
Source: J Clin Microbiol. 2025 Oct 9;63(11):e01050-25. doi: 10.1128/jcm.01050-25 (PMC12607571; doi:10.1128/jcm.01050-25)
Supplement: Table S1 — Antimicrobial agents and corresponding concentrations for the antimicrobial susceptibility testing assays studied. [file jcm.01050-25-s0001.pdf]

TABLE S1 Antimicrobial agents and corresponding concentrations for the antimicrobial susceptibility testing assays studied

Antimicrobial agent	VITEK REVEAL	VITEK 2-RAST	EUCAST reference BMD	DD-RAST disk	Antimicrobial powder suppliers for EUCAST reference BMD	Disk suppliers for DD-RAST
	Concentration range (µg/mL)			Content (µg)		
Amikacin	4–16	1–64	0.5–128	30	Sigma Aldrich	Oxoid Ltd
Amoxicillin/clavulanic acid	4/2–16/2	4/2–64/2	2/2–128/2	20/10	USP reference standard (amoxicillin)/ Sigma Aldrich (potassium clavulanate)	Oxoid Ltd
Ampicillin	4–8	1–64	0.5–128	10	Sigma Aldrich	Oxoid Ltd
Ampicillin/sulbactam	Not tested	2/4–32/4	1/4–64/4	Not tested	Sigma Aldrich/Sigma Aldrich	Not applicable
Aztreonam	1–16	1–64	0.5–128	Not tested	Sigma Aldrich	Not applicable
Cefepime	0.125–64	0.125–32	0.06–128	30	USP reference standard	Oxoid Ltd
Cefotaxime	0.125–4	0.25–64	0.06–128	5	Sigma Aldrich	Oxoid Ltd
Ceftazidime	0.125–64	0.125–64	0.06–128	10	Sigma Aldrich	Liofilchem S.r.l.
Ceftazidime/avibactam	0.25/4–16/4	0.125/4–16/4	0.06/4–32/4	10/4	Sigma Aldrich/Sigma Aldrich	Liofilchem S.r.l.
Ceftolozane/tazobactam	1/4–4/4	0.25/4–64/4	0.125/4–128/4	30/10	Sigma Aldrich/Sigma Aldrich	Oxoid ltd
Ceftriaxone	Not tested	0.25–64	0.125–128	Not tested	Sigma Aldrich	Not applicable
Ciprofloxacin	0.06–1	0.06–4	0.03–8	5	Sigma Aldrich	Oxoid Ltd
Eravacycline	Not tested	0.125–8	0.06–16	Not tested	MedChemExpress	Not applicable
Ertapenem	0.125–1	0.125–8	0.06–16	Not tested	Sigma Aldrich	Not applicable
Gentamicin	2–4	1–16	0.5–32	10	Sigma Aldrich	Oxoid Ltd
Imipenem	1–8	0.25–16	0.125–32	10	Sigma Aldrich	Oxoid Ltd
Imipenem/relebactam	Not tested	0.25/4–16/4	0.125/4–32/4	10/25	Sigma Aldrich (imipenem)/MedChemExpress (relebactam)	Liofilchem S.r.l.
Levofloxacin	0.25–1	0.125–8	0.06–16	5	Toku-E	Liofilchem
Meropenem	0.125–8	0.25–16	0.06–32	10	Sigma Aldrich	Oxoid Ltd
Meropenem/vaborbactam	2/8–8/8	0.5/8–64/8	0.25/8–128/8	20/10	Sigma Aldrich (meropenem)/ MedChemExpress (vaborbactam)	Oxoid Ltd
Piperacillin	8–16	Not tested	4–32	Not tested	Sigma Aldrich	Not applicable
Piperacillin/tazobactam	4/4–16/4	4/4–128/4	2/4–256/4	30/6	Sigma Aldrich/Sigma Aldrich	Oxoid Ltd
Tigecycline	0.5–1	0.5–8	0.25–16	Not tested	Sigma Aldrich	Not applicable
Tobramycin	2–4	1–16	0.5–32	10	Sigma Aldrich	Oxoid Ltd
Trimethoprim/sulfamethoxazole	2/38–4/76	1/19–16/304	0.5/9.5–32/608	1.25/23.75	Sigma Aldrich/Sigma Aldrich	Oxoid Ltd

Abbreviations: BMD, broth microdilution method; DD, disk diffusion; EUCAST, European Committee on Antimicrobial Susceptibility Testing; RAST, rapid antimicrobial susceptibility testing.
