# Supplementary material for: Improving time-to-result: head-to-head comparison of three rapid AST systems for Gram-negative bacteremia, including the newly developed VITEK REVEAL
Source: J Clin Microbiol. 2025 Oct 9;63(11):e01050-25. doi: 10.1128/jcm.01050-25 (PMC12607571; doi:10.1128/jcm.01050-25)
Supplement: Table S2 — Antimicrobial agents tested against Gram-negative bacterial species in the three RAST assays evaluated. [file jcm.01050-25-s0002.pdf]

TABLE S2 Antimicrobial agents tested against Gram-negative bacterial species in the three RAST assays evaluated

Antimicrobial agent	Bacterial species by VITEK REVEAL (R), VITEK 2-RAST (V), or DD-RAST (D) assay																	
	AB			AC			AL			CF			CK			ECC		
	R	V	D	R	V	D	R	V	D	R	V	D	R	V	D	R	V	D
Amikacin	■	■	■					■		■	■		■	■		■	■	
Amoxicillin/clavulanic acid													■	■			■	
Ampicillin																		
Ampicillin/sulbactam											■			■				
Aztreonam										■	■		■	■		■	■	
Cefepime					■					■	■		■	■		■	■	
Cefotaxime										■	■		■	■		■	■	
Ceftazidime										■	■		■	■		■	■	
Ceftazidime/avibactam										■	■			■		■	■	
Ceftolozane/tazobactam											■			■		■	■	
Ceftriaxone											■			■			■	
Ciprofloxacin	■	■	■		■			■		■	■		■	■		■	■	
Eravacycline																		
Ertapenem										■	■		■	■		■	■	
Gentamicin	■		■							■	■		■	■		■	■	
Imipenem	■	■	■					■		■	■		■	■		■	■	
Imipenem/relebactam											■			■			■	
Levofloxacin	■		■		■					■			■			■		
Meropenem	■	■	■					■		■	■		■	■		■	■	
Meropenem/vaborbactam										■	■		■	■		■	■	
Piperacillin										■						■		
Piperacillin/tazobactam										■	■		■	■		■	■	
Tigecycline													■					
Tobramycin	■	■	■							■	■		■	■		■	■	
Trimethoprim/sulfamethoxazole	■		■		■			■		■	■		■	■		■	■	

Antimicrobial agent	EC			KA			KO			KP			MM			PA		
	R	V	D	R	V	D	R	V	D	R	V	D	R	V	D	R	V	D
Amikacin	■	■	■	■	■		■	■		■	■	■		■		■	■	■
Amoxicillin/clavulanic acid	■	■	■		■		■	■		■	■	■		■				
Ampicillin	■	■	■		■			■			■							
Ampicillin/sulbactam		■						■			■							
Aztreonam	■	■		■	■		■	■		■	■			■		■	■	
Cefepime	■	■			■		■	■		■	■					■	■	■
Cefotaxime	■	■	■	■	■		■	■		■	■	■						
Ceftazidime	■	■	■	■	■		■	■		■	■	■		■		■	■	■
Ceftazidime/avibactam	■	■	■	■	■		■	■		■	■	■		■		■	■	■
Ceftolozane/tazobactam	■	■	■	■	■		■	■		■	■	■				■	■	■
Ceftriaxone		■			■			■			■							
Ciprofloxacin	■	■	■	■	■		■	■		■	■	■		■		■	■	■
Eravacycline		■																
Ertapenem	■	■		■	■		■	■		■	■			■				
Gentamicin	■	■	■	■	■		■	■		■	■	■		■				
Imipenem	■	■	■	■	■		■	■		■	■	■				■	■	■
Imipenem/relebactam		■	■		■			■			■	■					■	■
Levofloxacin	■		■	■			■			■		■						■
Meropenem	■	■	■	■	■		■	■		■	■	■		■		■	■	■
Meropenem/vaborbactam	■	■	■	■	■		■	■		■	■	■				■	■	■
Piperacillin	■			■			■			■						■		
Piperacillin/tazobactam	■	■	■	■	■		■	■		■	■	■		■		■	■	■
Tigecycline	■																	
Tobramycin	■	■	■	■	■		■	■		■	■	■		■		■	■	■
Trimethoprim/sulfamethoxazole	■	■	■	■	■		■	■		■	■	■		■				

Antimicrobial agent	PM			PS			RO			STM			SM			SE		
	R	V	D	R	V	D	R	V	D	R	V	D	R	V	D	R	V	D
Amikacin	■				■			■									■	
Amoxicillin/clavulanic acid	■	■			■			■									■	
Ampicillin	■	■																
Ampicillin/sulbactam		■																
Aztreonam		■			■			■						■			■	
Cefepime	■	■																
Cefotaxime	■	■																
Ceftazidime	■	■			■			■						■			■	
Ceftazidime/avibactam	■	■			■			■						■			■	
Ceftolozane/tazobactam		■																
Ceftriaxone		■																
Ciprofloxacin	■	■			■			■						■			■	
Eravacycline																		
Ertapenem	■	■			■			■						■			■	
Gentamicin	■	■						■						■				
Imipenem								■									■	
Imipenem/relebactam								■									■	
Levofloxacin	■																	
Meropenem	■	■			■			■						■			■	
Meropenem/vaborbactam	■				■			■						■			■	
Piperacillin	■																	
Piperacillin/tazobactam	■	■			■												■	
Tigecycline																		
Tobramycin	■	■						■										
Trimethoprim/sulfamethoxazole	■	■			■			■			■			■			■	

This table lists the Gram-negative bacterial species (n = 18) tested against each antimicrobial agent using the VITEK REVEAL (R), VITEK 2-RAST (V), and DD-RAST (D) assays. Colored cells indicate that the corresponding organism/antimicrobial agent combination was tested in the indicated assay; white cells indicate that it was not tested. When considering only the species tested by all three assays (*A. baumannii*, *E. coli*, *K. pneumoniae*, and *P. aeruginosa*), a total of 164 organisms were tested across 5, 15, 14, and 11 agent combinations, respectively. Of the 25 antimicrobial agents included in the study, eravacycline was tested only with the V assay against *E. coli*, whereas amikacin, ciprofloxacin, imipenem, meropenem, and tobramycin were tested with all R, V, and D assays across all four species.

Abbreviations: AB, *Acinetobacter baumannii*; AC, *Aeromonas caviae*; AL, *Acinetobacter lwoffii*; CF, *Citrobacter freundii*; CK, *Citrobacter koseri*; ECC, *Enterobacter cloacae* complex; EC, *Escherichia coli*; KA, *Klebsiella aerogenes*; KO, *Klebsiella oxytoca*; KP, *Klebsiella pneumoniae*; MM, *Morganella morganii*; PA, *Pseudomonas aeruginosa*; PM, *Proteus mirabilis*; PS, *Providencia stuartii*; RO, *Raoultella ornithinolytica*; STM, *Stenotrophomonas maltophilia*; SM, *Serratia marcescens*; SE, *Salmonella* enterica Typhimurium.
